# Keratinocyte Growth Factor Improves Epithelial Structure and Function in a Mouse Model of Intestinal Ischemia/Reperfusion

**DOI:** 10.1371/journal.pone.0044772

**Published:** 2012-09-13

**Authors:** Yujiao Cai, Wensheng Wang, Hongying Liang, Lihua Sun, Daniel H. Teitelbaum, Hua Yang

**Affiliations:** 1 Department of General Surgery, Xinqiao Hospital, Third Military Medical University, Chongqing, China; 2 Department of Surgery, the University of Michigan Medical School, Ann Arbor, Michigan, United States of America; University College London, United Kingdom

## Abstract

**Background:**

Intestinal ischemia/reperfusion (I/R) induces the desquamation of the intestinal epithelium, increases the intestinal permeability, and in patients often causes fatal conditions including sepsis and multiple organ failure. Keratinocyte growth factor (KGF) increases intestinal growth, although little is known about KGF activity on intestinal function after intestinal I/R. We hypothesized that KGF administration would improve the intestinal function in a mouse model of intestinal I/R.

**Methods:**

Adult C57BL/6J mice were randomized to three groups: Sham, I/R group and I/R+KGF group. Mice were killed on day 5, and the small bowel was harvested for histology, wet weight, RNA and protein content analysis. Epithelial cell (EC) proliferation was detected by immunohistochemistry for PCNA, and apoptosis was determined by TUNEL staining. The expressions of Claudin-1 and ZO-1 were detected by immunohistochemistry. Epithelial barrier function was assessed with transepithelial resistance (TER).

**Results:**

KGF significantly increased the intestinal wet weight, contents of intestinal protein and RNA, villus height, crypt depth and crypt cell proliferation, while KGF resulted in the decrease of epithelial apoptosis. KGF also stimulated the recovery of mucosal structures and attenuated the disrupted distribution of TJ proteins. Moreover, KGF attenuated the intestinal I/R-induced decrease in TER and maintained the intestinal barrier function.

**Conclusion:**

KGF administration improves the epithelial structure and barrier function in a mouse model of intestinal I/R. This suggests that KGF may have clinical applicability.

## Introduction

Intestinal ischemia/reperfusion (I/R) injury occurs in a variety of important clinical conditions [1]–[2]. The pathophysiology of intestinal I/R injury includes direct cellular damage due to ischemia and delayed dysfunction and damage as a result of the activation of inflammatory pathways [1]. The intestinal damage that ensues includes injury to the mucosal structure and barrier function.

Keratinocyte Growth Factor (KGF), also known as FGF7, is a known mitogenic growth factor, and expressed by T-cell receptor γδ intraepithelial lymphocytes (IEL) in the mucosal layer [3]. KGF receptors (KGFR) have been detected in high numbers in the gastrointestinal tract, which indicates that the gut can both synthesize and respond to KGF [4]–[6]. It is believed that KGF plays a critical role in the intestinal epithelial growth and maintenance. Studies have shown that KGF has a number of beneficial effects. A study from our group indicated that recombinant human KGF (rHuKGF) significantly improves the epithelial barrier function in a mouse model of total parenteral nutrition (TPN) [7]. In a mouse model of short bowel syndrome (SBS), 55% of the mid-small intestine was removed, and our results revealed IEL-derived KGF was significantly increased [8]. Studies have demonstrated that exogenous KGF could ameliorate the mucosal injury in several colitis animal models [9]–[11], while KGF null mice were more susceptible to dextran sulfate-induced colonic injury than their wild-type counterparts, and in the absence of KGF, healing was delayed [12]. Moreover, a study showed rHuKGF could increase the gas exchange and improve the mechanics of the lung in the oleic acid (OA) induced lung injury [13]. All these results suggest that KGF plays a critical role in the intestinal mucosal protection and repair in chronic intestinal injury. However, the effects of KGF on the intestinal mucosa in the ischemia/reperfusion (I/R) induced acute intestinal injury remain unclear. In the present study, we hypothesized that KGF could attenuate the damage to the epithelial structure and function in a mouse model of intestinal I/R.

## Materials and Methods

### Animals

Studies reported here conformed to the guidelines for the care and use of laboratory animals established by the University Committee on Use and Care of Animals at the Third Military Medical University, and the whole protocol was approved by this committee. Male, 6–8-week-old, specific pathogen-free, C57Bl/6 mice were purchased from the Laboratory Animal Center (Third Military Medical University, Chongqing, P.R. China), maintained in temperature, humidity and light-controlled conditions, and then randomized into sham group, I/R group and I/R+KGF group. Recombinant human KGF rHuKGF was intraperitoneally given in the I/R+KGF group at 5 mg/kg/day once daily for 5 days before the operation.

### Intestinal ischemia-reperfusion injury

After intraperitoneal anesthesia with 40 mg/kg pentobarbital, the abdomen was opened at the midline, and the superior mesenteric artery (SMA) was occluded for 20 min using non-traumatic vascular clamps, followed by reperfusion for 0 h, 6 h, 24 h and 72 h. Animals in the sham group underwent identical procedures without SMA occlusion (Sham). At the end of surgery, mice were given *ad libitum* access to water and food. There was no significant difference in the survival between the I/R+KGF group and the I/R group. In all experiments, at least 6 animals were included in each group and experiment was repeated at least three times.

### Histological examination

Segments of jejunum were harvested, fixed in 4% paraformaldehyde and used for histological examination. Tissues were dehydrated in the ethanol and embedded in the paraffin. Sections were obtained for hematoxylin-eosin (H&E) staining. The intestinal mucosal injury was evaluated under a light microscope according to the criteria described by Chiu et al [14] and graded from 0 to 5. Histological findings were assessed and scored by a pathologist blind to the experiment. The villus height and the depth of crypt were measured using a calibrated micrometer. A mean of 7 different fields were selected for the measurement of villus height and crypt depth.

### Measurements of mucosal wet weight and content of RNA and protein

At the time of tissue harvest, 10 cm of jejunum was obtained, weighed and used for the measurement of intestinal RNA and protein content. Intestinal mucosal RNA was determined by spectrophotometry using a modified Schmidt-Tannhauser method as described by Munro and Fleck [15]. Protein determination was performed by using a Bio-Rad protein assay kit (Bio-Rad Laboratories, Hercules, CA). RNA is expressed in µg/cm segment of intestine and proteins are expressed in mg/cm segment of intestine.

### TUNEL staining

TUNEL staining was performed according to the manufacturer's instructions (*In Situ* Cell Death Detection Kit; Roche, Germany). In brief, the paraffin-embedded sections were deparaffinized with xylene followed by absolute ethanol, 95% ethanol, 85% ethanol, 75% ethanol and 70% ethanol. The sections were washed with phosphate-buffered saline (PBS) for 5 min and treated with protease K (20 µg/mL) for 20 min at room temperature. After washing in PBS twice, these sections were incubated with 50 µl of TUNEL reaction solution in a humidified environment at 37°C for 60 min. Following washing in PBS thrice, these sections were treated with 50 µl of converter-POD in a humidified environment at 37°C for 30 min. Following washing in PBS thrice, sections were incubated with 100 µl of substrate at room temperature for 10 min. Mounting was performed after washing in PBS thrice. The number of TUNEL positive cells was counted under a microscope (400×), and the apoptotic cells in each section were counted in 7 independent fields followed by averaging.

### Western blot assay

Proteins were extracted from the intestinal mucosa followed by homogenization by sonication and centrifugation at 14,000 rpm for 15 min at 4°C. Twenty-five micrograms of protein were separated on the 10% SDS polyacrylamide gel and transferred to 0.2-µm nitrocellulose membrane which was then blocked in TBST (10 mM Tris-HCl, pH 7.5, 150 mM NaCl, and 0.1% Tween 20) containing 5% milk for 1 h. Blots were incubated with anti-KGF antibody (catalogue no.bs-0734R; Beijing Biosynthesis Biotechnology Co., Ltd.) overnight at 4°C. After washing, the membrane was incubated with HRP-conjugated secondary antibodies (Cell Signaling) and then visualized with an enhanced chemiluminescence (Cell Signaling). β-tubulin (Sigma, Dorset, UK) was used as an internal control. Optical density (OD) was determined, and OD of target protein was normalized by that of β-tubulin and presented as percentage of control.

### Detection of Epithelial Proliferation

Tissues were fixed in 4% paraformaldehyde and cut into 5-µm sections which were treated with 0.5% hydrogen peroxide in methanol, blocked for 45 min, and then incubated with anti-PCNA antibody (catalogue no.10205-2-AP; Proteinteck) or purified rabbit IgG (10 mg/ml; negative control) overnight at 4°C. These sections were incubated with biotinylated goat anti-rabbit IgG for 60 min, and then with streptavidin-enzyme conjugate (Vector Laboratories Inc). The peroxidase activities were developed with diaminobenzidine (DAB). After counterstaining with hematoxylin, histological examination was done under a light microscope (400×).

The crypt cell proliferation rate was calculated as the ratio of the number of crypt cells positive for PCNA to the total number of crypt cells. The total number of proliferating cells per crypt was defined as a mean of proliferating cells in 10 crypts.

### Immunohistochemistry

Anti-Claudin-1 antibody (catalogue no.ab-15098; Abcam Inc.) and anti-ZO-1 antibody (catalogue no.21773-1-AP; Proteinteck) were used for immunohistochemistry staining as described above. After counterstaining with hematoxylin, the localization of Claudin-1 and ZO-1 was examined under light microscope(630×).

### Detection of intestinal permeability

The transmembrane resistance (TER, Ω.cm^2^), an indicator of intestinal epithelial barrier function and tissue viability [16], was determined using the Ohm's law. The permeability of the small intestine was evaluated by TER. Tissues were bathed on the serosal and mucosal sides with Ringer solution. Bathing solutions were oxygenated (95% O_2_-5% CO_2_) and circulated in water-jacketed reservoirs at 37°C. After a 20-min equilibration period in Ussing chambers, detection was done for up to 1.5 h. TER was calculated from the spontaneous potential difference (PD) and short-circuit current.

### Statistical analysis

Data are expressed as mean ± standard deviation (SD). Statistics were performed using SPSS version 13.0 for windows. Results were analyzed using analysis of variance (ANOVA). A value of *P*<0.05 was considered statistically significant.

## Results

### Changes in KGF expression in a mouse model of acute intestinal I/R

**Figure 1:**
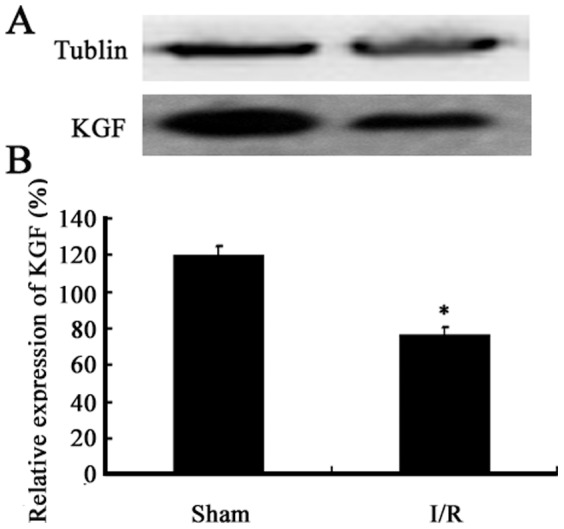
Change in KGF expression in a mouse model of acute intestinal I/R. (A) Decreased expression of KGF was confirmed by western blot assay in mice early after acute intestinal I/R, when compared with the sham group. Tubulin was used as an internal control. (B) Spearman rank correlation of KGF protein level with acute intestinal I/R in mice. *P<0.05 vs sham group, n = 6 per group.

Western blot assay was performed to detect the KGF expression. Results showed that the KGF expression significantly decreased by 35.9±4.5% early after intestinal I/R when compared with the sham group (P<0.05) (Figure 1). This suggests that KGF involves in the intestinal mucosal injury after intestinal I/R.

### Morphological examination of intestine

**Figure 2:**
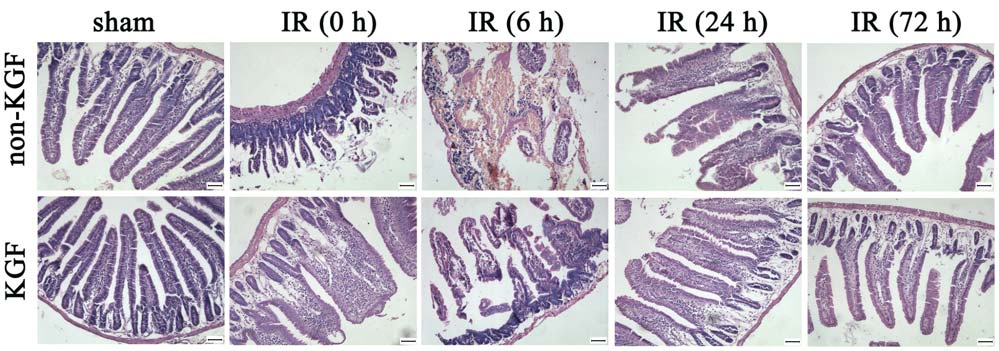
Histological changes in intestinal I/R mice with KGF treatment by H&E staining. More severe mucosal injury was observed in I/R group than in I/R+KGF group, while no mucosal injury was noted in the sham group; Original magnification: ×200, n = 6 per group. Scale bar = 50 µm.

Animals were sacrificed at 0 h, 6 h, 24 h and 72 h after surgery. The results in histological examination and classification of the lesions are summarized in Table 1. Results showed that there were no lesions in the intestinal mucosa in the sham group, whereas the intestine of I/R group (6 h) presented with grade 5 and 4 injuries of the intestinal mucosa, which were more severe than those in the I/R (6 h) +KGF group, characterized by the loss of villi or epithelial shedding from the villi. Grade 2 and 3 injuries were observed in the I/R (6 h) +KGF group, with partly epithelial lifting down the sides of the villi (Figure 2).

**Table 1:** Classification of mucosa lesions in the experimental animals*.

Animals	Mucosal lesion severity		
	Sham group	I/R group	I/R+ KGF group
1	0	4	2
2	0	4	3
3	0	5	2
4	0	4	3
5	0	5	2
6	0	4	2

* Tissues were harvested at 6 h after intestinal I/R.

### Intestinal wet weight and content of RNA and protein

As KGF markedly improved the intestinal morphology at 6 h after I/R, this time point was chosen for the following experiments. The mucosal wet weight of the jejunum was significantly increased in the I/R (6 h) +KGF group (561.3±10.2 mg/10 cm) when compared with the I/R group (405.7±8.9 mg/10 cm) at 6 h after intestinal I/R (P<0.05) (Table 2). As shown in Table 2, there was significant difference in the RNA content between the I/R (6 h)+KGF group (37.5±5.4 µg/cm) and the I/R group (21.2±4.3 µg/cm) at 6 h after intestinal I/R (P<0.05). The changes in the protein content of jejunum mucosa were similar to those in RNA content, and significant difference was also noted between the I/R (6 h) +KGF group (2.57±0.14 mg/cm) and the I/R (6 h) group (1.84±0.29 mg/cm) (P<0.05) (Table 2).

**Table 2:** Intestinal wet weight and contents of jejunal protein and RNA#.

	Sham	I/R group	I/R+ KGF group
Protein (mg/cm)	1.72±0.21	1.84±0.29	2.57±0.14*
RNA (µg/cm)	14.6±2.3	21.2±4.3	37.5±5.4*
Intestinal wet weight (mg/10 cm)	392.5±7.3	405.7±8.9	561.3±10.2*

# Tissues were harvested at 6 h after intestinal I/R,

* *P*<0.05 vs I/R group.

### Intestinal epithelial proliferation

**Figure 3:**
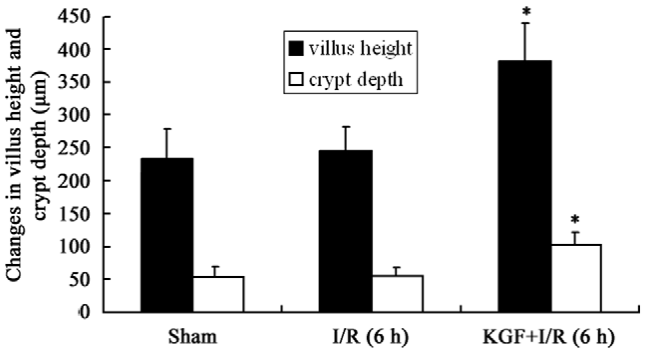
Alterations in villus height and crypt depth in mice after KGF treatment. * *P*<0.05 vs. I/R (6 h) group, n = 6 per group.

There was an increase in both villus height and crypt depth after KGF treatment. KGF treatment led to a significant increase in the jejunal villus height (382±62 µm) when compared with the I/R (6 h) group (247±41 µm) (P<0.05). The crypt depth was also significantly greater in the I/R (6 h) +KGF group (103±18 µm) than that in the I/R group (56±12 µm) at 6 h after intestinal I/R (P<0.05) (Figure 2, 3).

**Figure 4:**
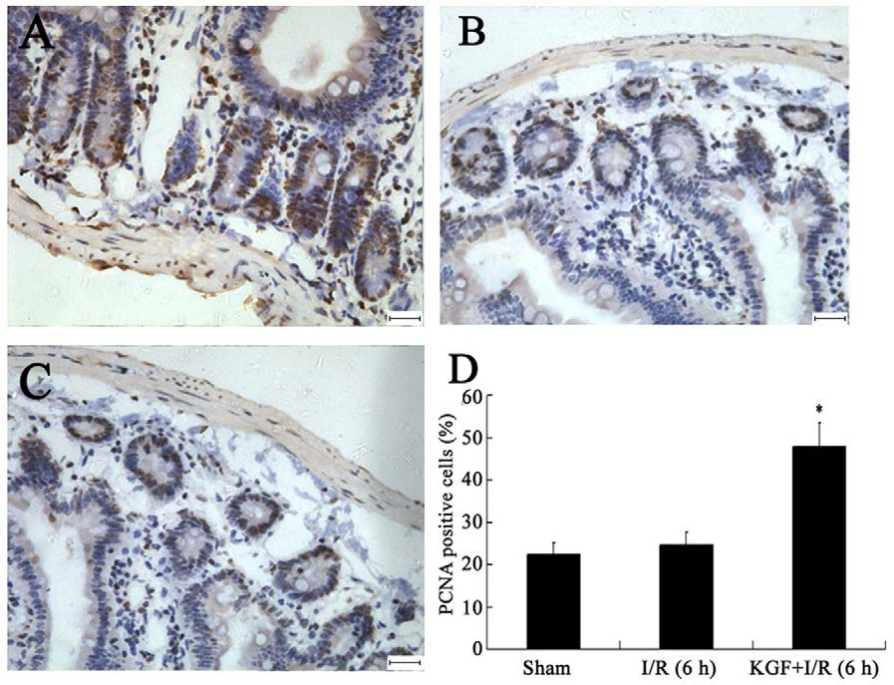
Alterations in PCNA expression in small intestine of KGF treated mice by immunohistochemistry. PCNA expression was significantly increased in I/R+KGF (6 h) group (A), as compared to the I/R (6 h) group (B) and sham group (C). Original magnification: ×400; (D) PCNA expression is expressed as means ± SD, *P<0.05 vs I/R group, n = 6 per group. Scale bar = 25 µm.

PCNA-positive cells were all distributed in the crypt of Lieberkuhn of the small intestine. In addition, KGF significantly increased the number of PCNA positive cells in the I/R (6 h)+KGF group (47.9±5.6%) when compared with the I/R group (24.6±3.1%) at 6 h after I/R (P<0.05) (Figure 4). There was no difference in the location of PCNA positive cells between groups, and all positive cells were found in the crypts.

### Intestinal epithelial apoptosis

**Figure 5:**
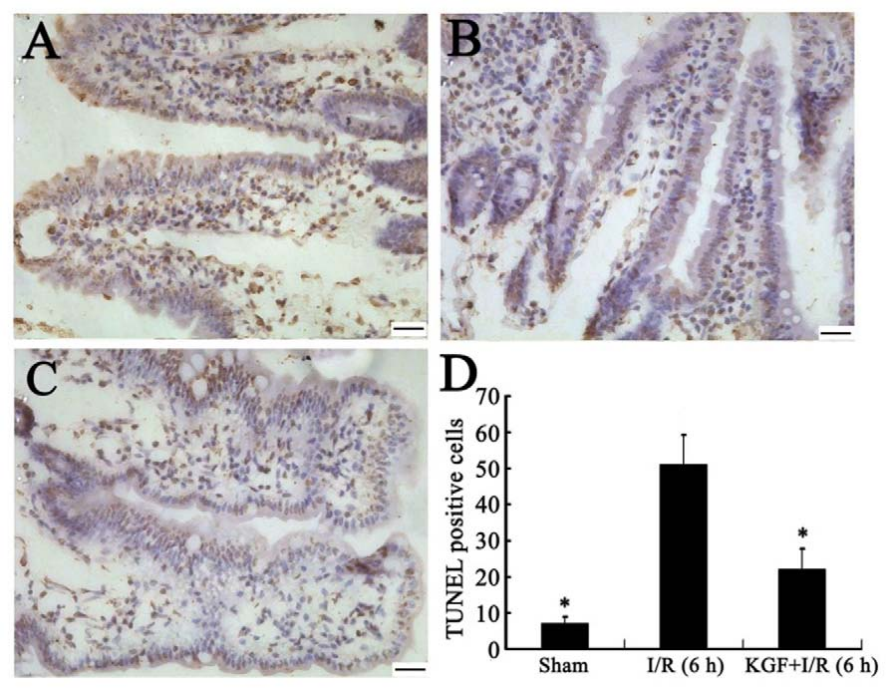
TUNEL staining of small intestine of mice after KGF treatment. The number of TUNEL positive cells was counted at a high magnification (×400). The number of TUNEL positive cells was significantly increased in the I/R group after 6 h intestinal I/R (A), as compared to the sham group (B); KGF significantly prevented the increase in TUNEL positive cells in the I/R(6 h)+KGF group (C) when compared with the I/R (6 h) group; (D) The number TUNEL positive cells is presented as mean ± SD. * *P*<0.05 vs. I/R group, n = 6 per group. Scale bar = 25 µm.

To determine whether KGF could attenuate the increase of intestinal epithelial apoptosis due to I/R, TUNEL staining was performed. Results showed that the highest amount of TUNEL positive cells was noted at 6 h after intestinal I/R (51±8.3%) when compared with the sham group (7±1.6%) (P<0.05) (Figure 5), but KGF significantly attenuated the increase in TUNEL-positive cells (22±5.7%) when compared with the I/R (6 h) group (51±8.3%) (P<0.05) (Figure 5). These findings suggest that KGF could attenuate the increase in I/R-induced intestinal epithelial apoptosis.

### Changes in expression of tight junction proteins

**Figure 6:**
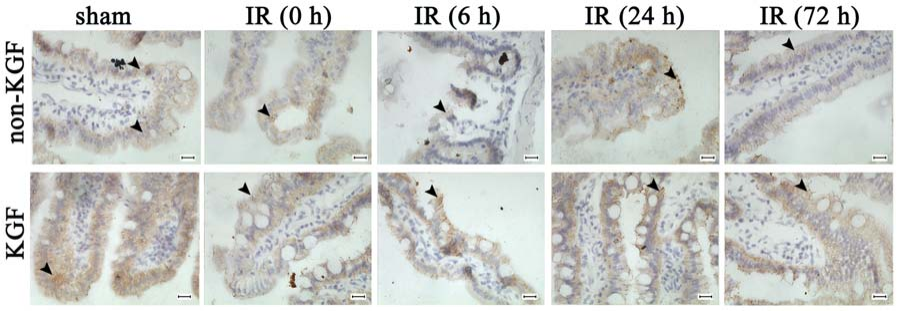
Changes in Claudin-1 expression in acute intestinal I/R mice after KGF treatment by immunohistochemistry. Claudin-1 expression was detected at 0 h, 6 h, 24 h and 72 h after I/R with or without KGF treatment, compared with the sham group. The reticular structures were significantly improved and maintained after KGF treatment at all stages of I/R injury when compared with the sham group. Arrow, Claudin-1 expression. Original magnification: ×630, n = 6 per group. Scale bar = 25 µm.

**Figure 7:**
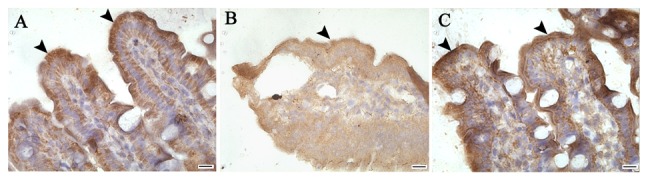
Changes in ZO-1 expression in acute intestinal I/R mice after KGF treatment by immunohistochemistry. ZO-1 expression was detected in the sham group (A), at 6 h after I/R (B) and after KGF treatment (C). KGF maintained the intense apical staining for ZO-1 at 6 h after I/R when compared with the I/R (6 h) group. Arrow, ZO-1 expression. Original magnification: ×630, n = 6 per group. Scale bar = 25 µm.

It has been found that tight junction (TJ) proteins are critical structural proteins in the maintenance of mucosal barrier function [17]–[18]. The impairment of mucosal barrier function is directly characterized by the aberrant expression of TJ proteins [17]–[19]. Immunohistochemistry was used to detect the expression of TJ proteins including Claudin-1 and ZO-1. The expression of Claudin-1 at 6 h and 24 h after intestinal I/R showed a significantly disrupted, diffuse staining pattern, while the normal chicken wire pattern was seen in the cross sections of the sham group (Figure 6). However, KGF improved the abnormal distribution of Claudin-1 following I/R, the reticular structures were more apparent and favorably maintained, especially in the I/R+KGF at 6 h or 24 h after intestinal I/R when compared with the I/R group at the corresponding time points (Figure 6). The ZO-1 exclusively localizes at the TJ of villous enterocytes, and an intense apical staining was found in the sham group (Figure 7). The apical staining of ZO-1 was obviously interrupted and partly disappeared in the I/R group, as compared to the sham group at 6 h after intestinal I/R , while the subtotal structure of ZO-1 was remained in the I/R (6 h)+KGF group, which was similar to the changes in the Claudin-1 expression (Figure 7). These results suggest that KGF can exert protective effect on the TJ proteins after acute intestinal I/R injury.

### Transepithelial resistance

**Figure 8:**
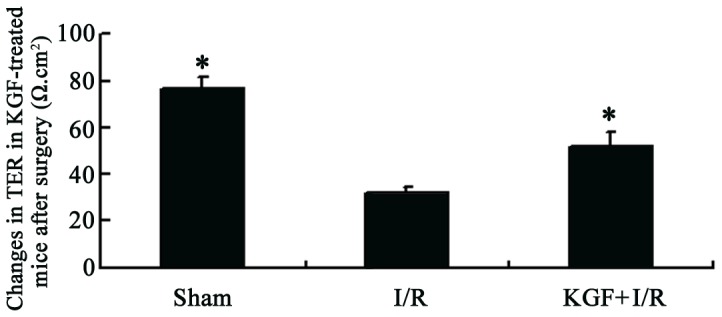
Changes in TER of I/R mice after KGF treatment. The permeability of the small intestine was evaluated by TER. Tissues were harvested at 6 h after intestinal I/R, *P<0.05 vs. I/R group, n = 6 per group.

To further confirm the role of KGF in the maintenance of intestinal barrier function, transepithelial resistance (TER) was detected with Ussing chambers [16]. Because our study revealed that more severe destruction was found in the intestinal morphology, and the location of TJ proteins was disrupted at 6 h after acute intestinal injury, the intestinal barrier function was determined at 6 h after surgery. The baseline TER (0 min after mounting) was 76.6±5.2 Ω.cm^2^ and 31.7±2.8 Ω.cm^2^ in the sham group and I/R (6 h) group, respectively. I/R resulted in a significant decrease in the TER (58.6%) when compared with the sham group. KGF partially attenuated the decrease in TER following intestinal I/R (26.1%), although the TER remained lower than that in the sham group (Figure 8). All these findings suggest that KGF can significantly attenuate the I/R-induced epithelial barrier dysfunction.

## Discussion

In this study, the effects of rHuKGF on the intestinal epithelial structure, function and growth were investigated in a mouse model of acute intestinal I/R. Our results demonstrated that KGF could exert protective effects in preventing the loss of epithelial structure and barrier function after acute intestinal I/R. Results also revealed KGF significantly improved the content of intestinal RNA, protein, the mucosal wet weight, increased the number of proliferating cells in the crypt, while decreased the intestinal epithelial apoptosis.

Intestinal I/R injury is a significant clinical problem arising from diseases or as a consequence of abdominal surgery [1]. A number of changes may occur in the intestinal mucosa after acute intestinal I/R, including a decline in the mucosal epithelial growth and function. Damage to the mucosa includes the epithelial shedding, bacterial translocation, disordered mucosal permeability, and alteration of the absorptive function [20]–[21]. In the present study, the histological features occurring in the I/R group were characterized by the shortening of the villi, loss of villous epithelium, multiple erosions, inflammatory cell infiltration, necrosis and hemorrhage into the intestinal wall. Our findings were consistent with those in previous studies on intestinal I/R in animals [22]–[23]. Our results indicated that the mucosal lesions (grade 4, 5) were more severe at 6 h after intestinal I/R and KGF significantly decreased the mucosal lesions (grade 2, 3), which suggested that KGF could improve the mucosal morphology in a mouse model of acute intestinal I/R.

The regulation of gastrointestinal cell growth and differentiation is complex and usually influenced by many factors [24]. KGF has been shown to regulate the proliferative response of the intestinal tract [25], which supports that KGF plays an important role in the intestinal epithelial growth and its maintenance. Studies have shown that KGF (rHuKGF) can improve the survival, body weight loss, hematochezia, diarrhea, and the histopathology in a dextran sodium sulfate-induced inflammatory bowel disease (IBD) mouse model [9], [26]. Exogenous KGF can prevent the mucositis caused by chemotherapy and radiation to the intestine [27]. KGF has also been shown to ameliorate the mucosal injury in an experimental model of intestinal inflammation in rats [9], [11]. All these results suggest that KGF plays a critical role in the intestinal mucosal protection and repair in the chronic intestinal injury, which was also confirmed in a mouse model of acute intestinal I/R in our study.

In the present study, results showed that KGF expression significantly decreased in mice early after acute intestinal I/R, which suggested that KGF was involved in the acute intestinal I/R in mice. However, exogenous rHuKGF increased the PCNA-positive epithelial cells, villus height and crypt depth and attenuated the changes in the mucosal morphology. The mucosal wet weight, the content of intestinal RNA and protein were also found significantly increased by exogenous KGF in our study. A study from Johnson et al [28] showed that KGF significantly increased the villus height in a rat SBS model. The mucosal wet weight, the content DNA and protein in rats were also significantly increased by KGF at 3 days after an 85% small bowel resection in a rat model [28]. BrdU-positive epithelial cells further increased in the SBS group after KGF treatment when compared with the non-treated SBS group [8]. Our previous study also revealed that KGF significantly increased the intestinal wet weight, the content of intestinal DNA and protein in the TPN mice, and led to an increase in the villus height and crypt cell proliferation [7]. Additionally, our results revealed that KGF significantly reduced the intestinal epithelial apoptosis of the small intestine, although I/R actually induced the epithelial apoptosis and mucosal injury in the mouse intestine, which was consistent with findings in the study of Hung et al [29]. Taken together, our results showed that KGF could affect either the proliferation or apoptosis of the intestinal epithelial cells in the mice with intestinal I/R, and KGF may play an important role in this pathological condition.

TJs are the major determinants of paracellular permeability. Intestinal injury alters the distribution of TJ proteins *in vivo*, which is associated with the functional TJ (barrier function) deficiency. Although the changes in the epithelial TJ protein expression have been studied extensively in monolayers stimulated by cytokines, bacteria, or aspirin [30]–[35], much remains to be understood about the changes in the barrier function following acute intestinal I/R *in vivo*. In the present study, I/R resulted in the disruption of TJ proteins (Claudin-1 and ZO-1), which was consistent with previous findings [19], [36]–[37]. The gut permits the absorption of nutrients while prevents the systemic contamination by luminal toxins and microbial products [38]. TER has been used to detect the tissue TJ integrity. Our results showed that the intestinal I/R caused an increase in the intestinal permeability. This result was supported by the findings of Higuchi et al [39], in which I/R in a rat model led to an increased intestinal conductance.

In the present study, TJ proteins including Claudin-1 and ZO-1 were damaged by intestinal I/R, KGF could attenuate this damages to TJ proteins. KGF also partly attenuated the intestinal barrier dysfunction after intestinal I/R. All these findings further confirmed that KGF played an important role in the maintenance of intestinal barrier function in acute intestinal I/R. This protective effect of KGF was also observed in our previous study in a mouse TPN model, in which KGF significantly attenuated the barrier dysfunction due to TPN [7]. However, the mechanism underlying the protective effect of KGF on the intestinal integrity is still poorly understood. Our findings provide evidence that KGF attenuates the disrupted distribution of TJ proteins, which may partially improve the functional TJ deficiency.

In conclusion, this study demonstrated that intestinal I/R caused profound changes in the small bowel epithelial morphology and physiology in a mouse model. These changes included the mucosal injury and the disruption in the TJ protein expression and the alteration in the epithelial barrier function. Moreover, KGF significantly attenuated these changes. These results demonstrate that KGF can exert protective effects to prevent the loss of epithelial structure and barrier function after acute intestinal I/R.

## References

[1] Mallick IH, Yang W, Winslet MC, Seifalian AM (2004) Ischemia reperfusion injury of the intestine and protective strategies against injury. Dig Dis Sci 49 9 1359–77. 15481305 10.1023/b:ddas.0000042232.98927.91

[2] Koçogullari CU, Becit N, Erkut B, Keleş MS, Ceviz M, et al (2008) Prevention of reperfusion injury of the spinal cord in aortic surgery: an experimental study. Surg Today 38 3 237–44. 18306998 10.1007/s00595-007-3614-5

[3] Boismenu R, Havran WL (1994) Modulation of epithelial cell growth by intraepithelial gamma delta T cells. Science 266 5188 1253–5. 7973709 10.1126/science.7973709

[4] Finch PW, Rubin JS (2004) Keratinocyte growth factor/fibroblast growth factor 7, a homeostatic factor with therapeutic potential for epithelial protection and repair. Adv Cancer Res 91: 69–136. 15327889 10.1016/S0065-230X(04)91003-2

[5] Revest JM, Suniara RK, Kerr K, Owen JJ, Dickson C (2001) Development of the thymus requires signaling through the fibroblast growth factor receptor R2-IIIb. J Immunol 167 4 1954–61. 11489975 10.4049/jimmunol.167.4.1954

[6] Housley RM, Morris CF, Boyle W, Ring B, Biltz R, et al (1994) Keratinocyte growth factor induces proliferation of hepatocytes and epithelial cells throughout the rat gastrointestinal tract. J Clin Invest 94 5 1764–77. 7962522 10.1172/JCI117524 PMC294567

[7] Yang H, Wildhaber B, Tazuke Y, Teitelbaum DH (2002) 2002 Harry M. Vars research award: Keratinocyte growth factor stimulates the recovery of epithelial structure and function in a mouse model of total parenteral nutrition. JPEN 26 6 333–40; discussion 340–1. 10.1177/0148607102026006333 12405644

[8] Yang H, Wildhaber BE, Teitelbaum DH (2003) 2003 Harry M. Vars Research Award. Keratinocyte growth factor improves epithelial function after massive small bowel resection. JPEN 27 3 198–206; discussion 206–7. 10.1177/0148607103027003198 12757114

[9] Byrne FR, Farrell CL, Aranda R, Rex KL, Scully S, et al (2002) rHuKGF ameliorates symptoms in DSS and CD4tCD45rbHi T cell transfer mouse models of inflammatory bowel disease. Am J Physiol Gastrointest Liver Physiol 282 4 G690–701. 11897629 10.1152/ajpgi.00314.2001

[10] Egger B, Procaccino F, Sarosi I, Tolmos J, Büchler MW, et al (1999) Keratinocyte growth factor ameliorates dextran sodium sulfate colitis in mice. Dig Dis Sci 44 4 836–44. 10219846 10.1023/a:1026642715764

[11] Zeeh JM, Procaccino F, Hoffmann P, Aukerman SL, McRoberts JA, et al (1996) Keratinocyte growth factor ameliorates mucosal injury in an experimental model of colitis in rats. Gastroenterology 110 4 1077–83. 8612996 10.1053/gast.1996.v110.pm8612996

[12] Chen Y, Chou K, Fuchs E, Havran WL, Boismenu R (2002) Protection of the intestinal mucosa by intraepithelial gamma delta T cells. Proc Natl Acad Sci U S A 99 22 14338–43. 12376619 10.1073/pnas.212290499 PMC137885

[13] Ulrich K, Stern M, Goddard ME, Williams J, Zhu J, et al (2005) Keratinocyte growth factor therapy in murine oleic acid-induced acute lung injury. Am J Physiol Lung Cell Mol Physiol 288 6 L1179–92. 15681392 10.1152/ajplung.00450.2004

[14] Chiu CJ, McArdle AH, Brown R, Scott HJ, Gurd FN (1970) Intestinal mucosal lesion in low-flow states. I. A morphologic, hemodynamic and metabolic reappraisal. Arch Surg 101 4 478–83. 5457245 10.1001/archsurg.1970.01340280030009

[15] Munro HN, Fleck A (1969) Analysis of tissues and body fluids for nitrogenous constituents; in Munro HN (ed): Mammalian Protein Metabolism. New York, Academic Press. pp. 465–483.

[16] Smith PL (1996) Methods for evaluating intestinal permeability and metabolism in vitro. Pharm Biotechnol 8: 13–34. 8791802 10.1007/978-1-4899-1863-5_2

[17] Samak G, Suzuki T, Bhargava A, Rao RK (2010) c-Jun NH2-terminal kinase-2 mediates osmotic stress-induced tight junction disruption in the intestinal epithelium. Am J Physiol Gastrointest Liver Physiol 299 3 G572–84. 20595622 10.1152/ajpgi.00265.2010 PMC3774214

[18] Strauman MC, Harper JM, Harrington SM, Boll EJ, Nataro JP (2010) Enteroaggregative Escherichia coli disrupts epithelial cell tight junctions. Infect Immun 78 11 4958–64. 20823198 10.1128/IAI.00580-10 PMC2976312

[19] Anderson RC, Cookson AL, McNabb WC, Park Z, McCann MJ, et al (2010) Lactobacillus plantarum MB452 enhances the function of the intestinal barrier by increasing the expression levels of genes involved in tight junction formation. BMC Microbiol 10: 316. 21143932 10.1186/1471-2180-10-316 PMC3004893

[20] Granger DN, Korthuis RJ (1995) Physiologic mechanisms of postischemic tissue injury. Annu Rev Physiol 57: 311–32. 7778871 10.1146/annurev.ph.57.030195.001523

[21] Matthijsen RA, Derikx JP, Kuipers D, van Dam RM, Dejong CH, et al (2009) Enterocyte shedding and epithelial lining repair following ischemia of the human small intestine attenuate inflammation. PLoS One 4 9 e7045. 19753114 10.1371/journal.pone.0007045 PMC2737143

[22] Daniel RA, Cardoso VK, Góis E Jr, Parra RS, Garcia SB, et al (2011) Effect of hyperbaric oxygen therapy on the intestinal ischemia reperfusion injury. Acta Cir Bras 26 6 463–9. 22042109 10.1590/s0102-86502011000600010

[23] Yucel AF, Kanter M, Pergel A, Erboga M, Guzel A (2011) The role of curcumin on intestinal oxidative stress, cell proliferation and apoptosis after ischemia/reperfusion injury in rats. J Mol Histol 42 6 579–87. 21984066 10.1007/s10735-011-9364-0

[24] Drucker DJ (1997) Epithelial cell growth and differentiation. I. Intestinal growth factors. Am J Physiol 273 1 Pt 1 G3–6. 9252503 10.1152/ajpgi.1997.273.1.G3

[25] Dignass A, Lynch-Devaney K, Kindon H, Thim L, Podolsky DK (1994) Trefoil peptides promote epithelial migration through a transforming growth factor beta-independent pathway. J Clin Invest 94 1 376–83. 8040278 10.1172/JCI117332 PMC296319

[26] Finch PW, Pricolo V, Wu A, Finkelstein SD (1996) Increased expression of keratinocyte growth factor messenger RNA associated with inflammatory bowel disease. Gastroenterology 110 2 441–51. 8566591 10.1053/gast.1996.v110.pm8566591

[27] Farrell CL, Bready JV, Rex KL, Chen JN, DiPalma CR, et al (1998) Keratinocyte growth factor protects mice from chemotherapy and radiation-induced gastrointestinal injury and mortality. Cancer Res 58 5 933–9. 9500453

[28] Johnson WF, DiPalma CR, Ziegler TR, Scully S, Farrell CL (2000) Keratinocyte growth factor enhances early gut adaptation in a rat model of short bowel syndrome. Vet Surg 29 1 17–27. 10653491 10.1111/j.1532-950x.2000.00017.x

[29] Hung WT, Chen Y, Tseng SH, Li HL, Chen CK (2004) Fetal bovine serum suppresses apoptosis in the small intestine after total ischemia and reperfusion in mice. J Pediatr Surg 39 7 1077–83. 15213903 10.1016/j.jpedsurg.2004.03.047

[30] Bruewer M, Luegering A, Kucharzik T, Parkos CA, Madara JL, et al (2003) Proinflammatory cytokines disrupt epithelial barrier function by apoptosis-independent mechanisms. J Immunol 171 11 6164–72. 14634132 10.4049/jimmunol.171.11.6164

[31] Bruewer M, Utech M, Ivanov AI, Hopkins AM, Parkos CA, et al (2005) Interferon-gamma induces internalization of epithelial tight junction proteins via a macropinocytosis-like process. FASEB J 19 8 923–33. 15923402 10.1096/fj.04-3260com

[32] Montalto M, Maggiano N, Ricci R, Curigliano V, Santoro L, et al (2004) Lactobacillus acidophilus protects tight junctions from aspirin damage in HT-29 cells. Digestion 69 4 225–8. 15205571 10.1159/000079152

[33] Parassol N, Freitas M, Thoreux K, Dalmasso G, Bourdet-Sicard R, et al (2005) Lactobacillus casei DN-114 001 inhibits the increase in paracellular permeability of enteropathogenic Escherichia coli-infected T84 cells. Res Microbiol 156 2 256–62. 15748992 10.1016/j.resmic.2004.09.013

[34] Resta-Lenert S, Barrett KE (2003) Live probiotics protect intestinal epithelial cells from the effects of infection with enteroinvasive Escherichia coli (EIEC). Gut 52 7 988–97. 12801956 10.1136/gut.52.7.988 PMC1773702

[35] Zyrek AA, Cichon C, Helms S, Enders C, Sonnenborn U, et al (2007) Molecular mechanisms underlying the probiotic effects of Escherichia coli Nissle 1917 involve ZO-2 and PKCzeta redistribution resulting in tight junction and epithelial barrier repair. Cell Microbiol 9 3 804–16. 17087734 10.1111/j.1462-5822.2006.00836.x

[36] Moeser AJ, Nighot PK, Ryan KA, Simpson JE, Clarke LL, et al (2008) Mice lacking the Na+/H+ exchanger 2 have impaired recovery of intestinal barrier function. Am J Physiol Gastrointest Liver Physiol 295 4 G791–7. 18719001 10.1152/ajpgi.00538.2007 PMC4838133

[37] Li Q, Zhang Q, Wang C, Liu X, Qu L, et al (2009) Altered distribution of tight junction proteins after intestinal ischaemia/reperfusion injury in rats. J Cell Mol Med 13 9B 4061–76. 19929946 10.1111/j.1582-4934.2009.00975.x PMC4516553

[38] Aranow JS, Fink MP (1996) Determinants of intestinal barrier failure in critical illness. Br J Anaesth 77 1 71–81. 8703632 10.1093/bja/77.1.71

[39] Higuchi S, Wu R, Zhou M, Marini CP, Ravikumar TS, et al (2008) Gut hyperpermiability after ischemia and reperfusion: attenuation with adrenomedullin and its binding protein treatment. Int J Clin Exp Pathol 1 5 409–18. 18787625 PMC2480576

